# *Cirolana phuketensis*, a new species of marine isopod (Crustacea, Isopoda, Cirolanidae) from the Andaman Sea coast of Thailand

**DOI:** 10.3897/zookeys.695.13771

**Published:** 2017-09-04

**Authors:** Eknarin Rodcharoen, Niel L. Bruce, Pornsilp Pholpunthin

**Affiliations:** 1 Department of Aquatic Science, Faculty of Natural Resources, Prince of Songkla University, Hat Yai, Songkhla, Thailand 90112; 2 Museum of Tropical Queensland, Queensland Museum, Townsville, Australia; and Water Research Group, Unit for Environmental Sciences and Management, North-West University, Private Bag X6001, Potchefstroom 2520, South Africa; 3 Department of Biology, Faculty of Science, Prince of Songkla University, Hat Yai, Songkhla, Thailand 90112

**Keywords:** Isopoda, Cirolanidae, *Cirolana*, new species, the Andaman Sea, Thailand

## Abstract

*Cirolana phuketensis* **sp. n.** was collected from coral rubble from the Andaman sea coast of Thailand. *C. phuketensis* **sp. n.** is described and fully illustrated; *C. phuketensis* **sp. n.** can be recognized by the presence of transverse sutures on pereonites 2–4, pereonite 7 having three transverse sutures forming a nodulose ridge, antennula peduncle with articles 1 and 2 fully fused; pleotelson dorsal surface with 2 sub-median longitudinal carinae, each of which has one prominent tubercle, lateral margins weakly convex, and posterior margin narrow and rounded; 6 molariform robust setae pereopod 1 on inferior margin of merus and the penial openings are two low tubercles. A dichotomous key to species of *Cirolana* in Thailand is given.

## Introduction

Thailand lies in the tropical zone between Pacific Ocean and Indian Ocean. This region has high marine biodiversity (Briggs 2000, 2005; Briggs and Bowen 2013; Carpenter et al. 2011) but knowledge of marine crustaceans still remains minimal in the region, the non-decapod taxa having received relatively little attention (see Bruce et al. 2002). Since 2000 several new species and new records of marine amphipods (Ariyama et al. 2010; Wongkamhaeng et al. 2012a, b, 2013, 2014), and isopods (Bruce and Olesen 2002; Storey 2002; Svavarsson 2002; Svavarsson and Gísladóttir 2002; Rodcharoen et al. 2014, 2016) have been described.

The family Cirolanidae Dana, 1852 (superfamily Cirolanoidea, suborder Cymothoida following Brandt and Poore 2003), consists of 61 accepted genera and 497 species worldwide (Bruce and Schotte 2015). Forty-three species in twelve genera of Cirolanidae are known from South-East Asia (Nierstrasz 1931; Bruce and Olesen 2002; Sidabalok 2013; Rodcharoen et al. 2014, 2016, present study; Sidabalok and Bruce 2015, 2016, 2017a, b, c in press, and in prep; excluding nomina dubia and synonyms) Bruce (2004b, table 1) compared the diversity of Cirolanidae from different regions, and one can readily assess that, given the relative low level of research on Cirolanidae in South-East Asia the diversity is relatively high and will increase significantly with further research. At present the diversity of South-East Asian Cirolanidae is second only to that of the well-documented Queensland coast that has 16 genera and 65 species (Bruce 2004b, updated). Thailand itself has 18 species in eight genera.

Species of *Cirolana* Leach, 1818 primarily occupy marine and estuarine habitats, and the genus is the largest in the family (Bruce 1981, 1986; Brusca et al. 1995) with 136 named species and a worldwide distribution (Bruce and Schotte 2015). *Cirolana* is found in all oceans from tropical regions to temperate regions. Only *Cirolana mclaughlinae* Bruce & Brandt, 2006, from the Ross Sea, occurs in polar waters. The genus is most common and diverse in the tropics (Bruce 1981, 1986; Kensley and Schotte 1989, 2005; Brusca et al. 1995). Nine species of *Cirolana* have been recorded in Thailand. Of these, five species were reported from the Andaman Sea. Kensley (2001) listed the species known from the Indian Ocean, including the western coasts of Thailand. Bruce and Olesen (2002) reported four marine cirolanid species from Andaman Sea including two new species of *Cirolana*. Recently, Rodcharoen et al. (2016) reported four new species of *Cirolana* ‘*parva* group’ from Thailand two of which are from the Andaman Sea.

*Cirolana phuketensis* sp. n. is described from the Andaman coast of Thailand and a key of *Cirolana* species occurring in Thai coastal waters is provided.

## Materials and methods

### 2.1 Sampling and collection

**Figure 1.**
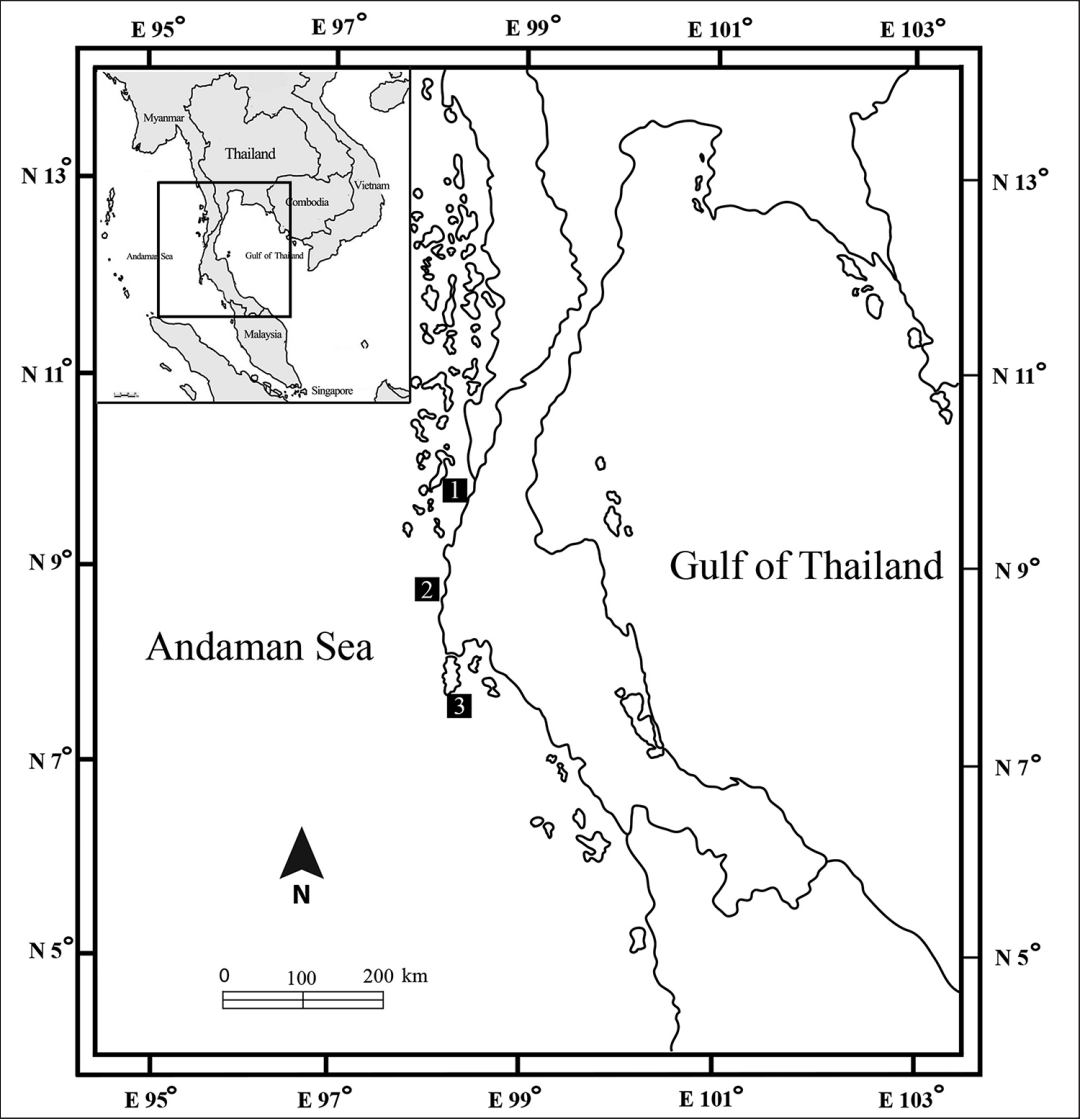
Map of sampling sites **1** Koh Phayam, Ranong Province **2** Laem Pakarang, Phang Nga Province **3** Ao Makham, Phuket Province.

Specimens were collected from shallow-water coral-rubble habitats (at depths of 0–10 m) in the coastal zone of the Andaman Sea (Figure 1) using baited traps as described by Keable (1995). Specimens were fixed in 10% formalin in the field and transferred to 70% ethanol.

### 2.2 Morphological study

Appendages of a paratype were dissected for description under Olympus SZ51 stereo microscope and drawn under an Olympus CH30 compound microscope with a *camera lucida*. The holotype dorsal and lateral drawings are based on photos taken by Olympus DP71 microscope digital camera with Olympus SZH10 stereo microscope. Drawings were inked using Adobe Illustrator with Wacom Bamboo drawing tablet. Morphological characters for the descriptions follow Bruce (2004a), and were prepared using DELTA (Descriptive Language for Taxonomy: Coleman et al. 2010; Dallwitz 1980; Dallwitz et al. 1997, 2006).

Abbreviations: **PSUZC**, Prince of Songkla University Zoological Collection; **MTQ**, Museum of Tropical Queensland. Queensland Museum; **PMS**, plumose marginal setae; **RS**, robust seta/setae; **CPS**, circumplumose setae.

## Taxonomy

### Family Cirolanidae Dana, 1952

#### Genus Cirolana

Taxon classificationAnimaliaIsopodaCirolanidae

Leach, 1818

##### Remarks.

For the most recent accounts of this genus in Thai waters see Bruce and Olesen (2002) and Rodcharoen et al (2014, 2016); Bruce and Wong (2015) and Sidabalok (2013) while not dealing with the Thai isopod fauna give a useful indication of the genera and species diversity that can be expected in the region. Diagnoses to *Cirolana* have been given most recently Brusca et al. (1995), Kensley and Schotte (1989).

#### Cirolana phuketensis sp. n.

Taxon classificationAnimaliaIsopodaCirolanidae

http://zoobank.org/CF35E681-77AD-47A1-B3FF-F1493DBDC6C5

##### Material examined.

**Holotype**: ♂ (5.0 mm), Ao Makham, Phuket Province, 07°49'51"N, 98°24'14"E, 6 August 2014, trapped in 1 m of depth, coral rubble, coll. E. Rodcharoen (PSUZC–CR2086-01).

Paratypes: 6 ♂ (4.6, 5.8, 5.2, 5.0, 5.3, 5.1 mm [dissected]), 3 ♀ (5.2, 4.9, 4.9 mm [dissected]), same data as holotype, (PSUZC–CR2086-02; MTQ W53037). 3 ♂ (5.6, 4.8, 5.3 mm [dissected]), 6 ♀ (5.2, 5.7, 5.3, 5.7, 5.6, 5.4 mm [dissected]), Laem Pakarang, Phang Nga Province, 08°44'11"N, 98°13'13"E, 15 march 2012, trapped in 2 m of depth, coral rubble, coll. E. Rodcharoen (PSUZC–CR2086-03; MTQ W53038). 3 ♂ (4.7, 4.9, 5.0 mm [dissected]), 7 ♀ (5.0, 4.7, 4.9, 4.9, 5.1, 4.6, 5.1 mm [dissected]), Koh Phayam, Ranong Province, 09°42'36"N, 98°23'41"E, 22 December 2012, trapped in 3 m of depth, coral rubble, coll. E. Rodcharoen (PSUZC–CR2086-04; MTQ W53039).

##### Description of male.

**Figure 2.**
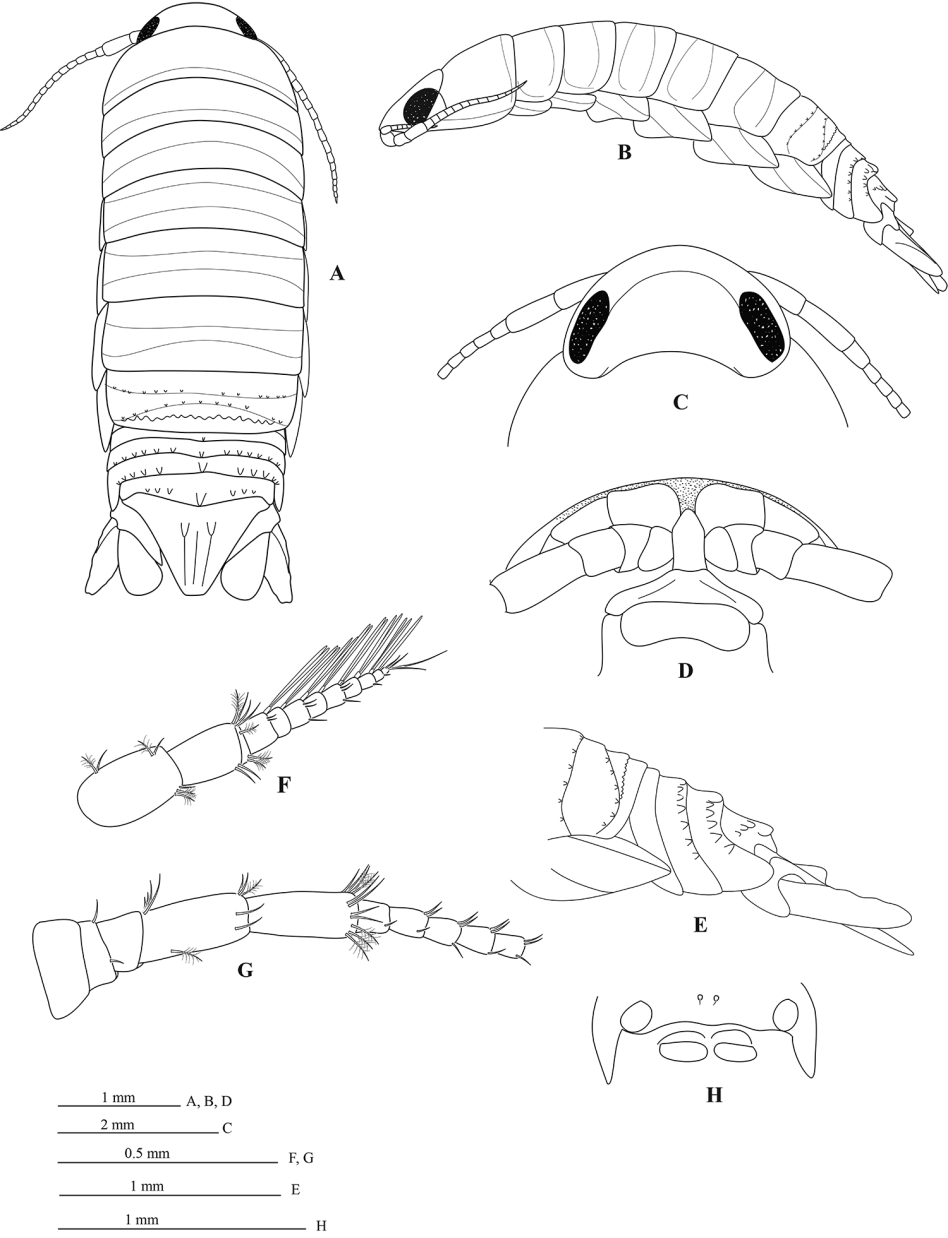
*Cirolana phuketensis* sp. n., male holotype, (PSUZC–CR0286-01) (5.0mm) (**A–E**), male paratype (PSUZC–CR0286-02) (5.1 mm) (**F–G**), male paratype (PSUZC–CR2086-04 (**H**) **A** dorsal view **B** lateral view **C** head **D** frons **E** pleon **F** antennula **G** antenna peduncle **H** penial opening, sternite 7.

*Body* 2.8 times as long as greatest width, widest at pereonite 6, lateral margins subparallel (Figure 2A). *Rostral point* absent (Figure 2C). *Eyes* colour dark brown (Figure 2C). *Pereonites* 2–4 with each a single transverse impressed suture; pereonites 5–6 with each 2 transverse impressed sutures; pereonite 7 with 3 transverse sutures each with a nodulose ridge (Figure 2A). *Pereonite 1 and coxae 2–3* (Figure 2B) each with posteroventral angle rounded; coxae 5–7 with entire oblique carina. *Pleon* (Figure 2E) with pleonite 1 largely concealed by pereonite 7; posterolateral angles of pleonite 2 forming acute point, extending posteriorly to anterior of pleonite 4; pleonite 3 with a row of 13 small tubercles, posterolateral margins not extending to posterior margin of pleonite 5, rounded; pleonite 4 with median tubercles and 5–6 sublateral tubercles on each side, posterolateral margin of pleonite 4 rounded, clearly extending beyond posterior margin of pleonite 5; pleonite 5 with prominent median tubercles and 3–4 sublateral tubercles on each side, posterolateral angles overlapped by lateral margins of pleonite 4. *Pleotelson* 0.7 times as long as anterior width, dorsal surface with 2 tubercles and paired submedian longitudinal carina; lateral margins weakly concave, margins serrate, posterior margin evenly rounded, without median point, with 6 robust setae interspersed among 10 slender plumose setae as figured (Figure 6C, D).

*Antennula* (Figure 2F) peduncle articles 1 and 2 entirely fused; articles 3 and 4 0.8 times as long as combined lengths of articles 1 and 2; article 3 1.6 times as long as wide, flagellum with 9 articles, antennula extending to anterior margin of pereonite 1. *Antenna* (Figure 2G) peduncle article 4 2.2 times as long as wide, 2.4 times as long as article 3, inferior margin with 1 plumose setae, inferodistal margin1 short simple setae; article 5 1.0 times as long as article 4, 2.4 times as long as wide, inferodistal angle with cluster of 3 pappose setae, anterodistal angle with cluster of 4 short simple setae and 2 plumose setae; flagellum with 16 articles, extending to middle of pereonite 4.

*Frontal lamina* (Figure 2D) pentagonal, lateral margins concave, anterior margin with narrowly round apex.

**Figure 3.**
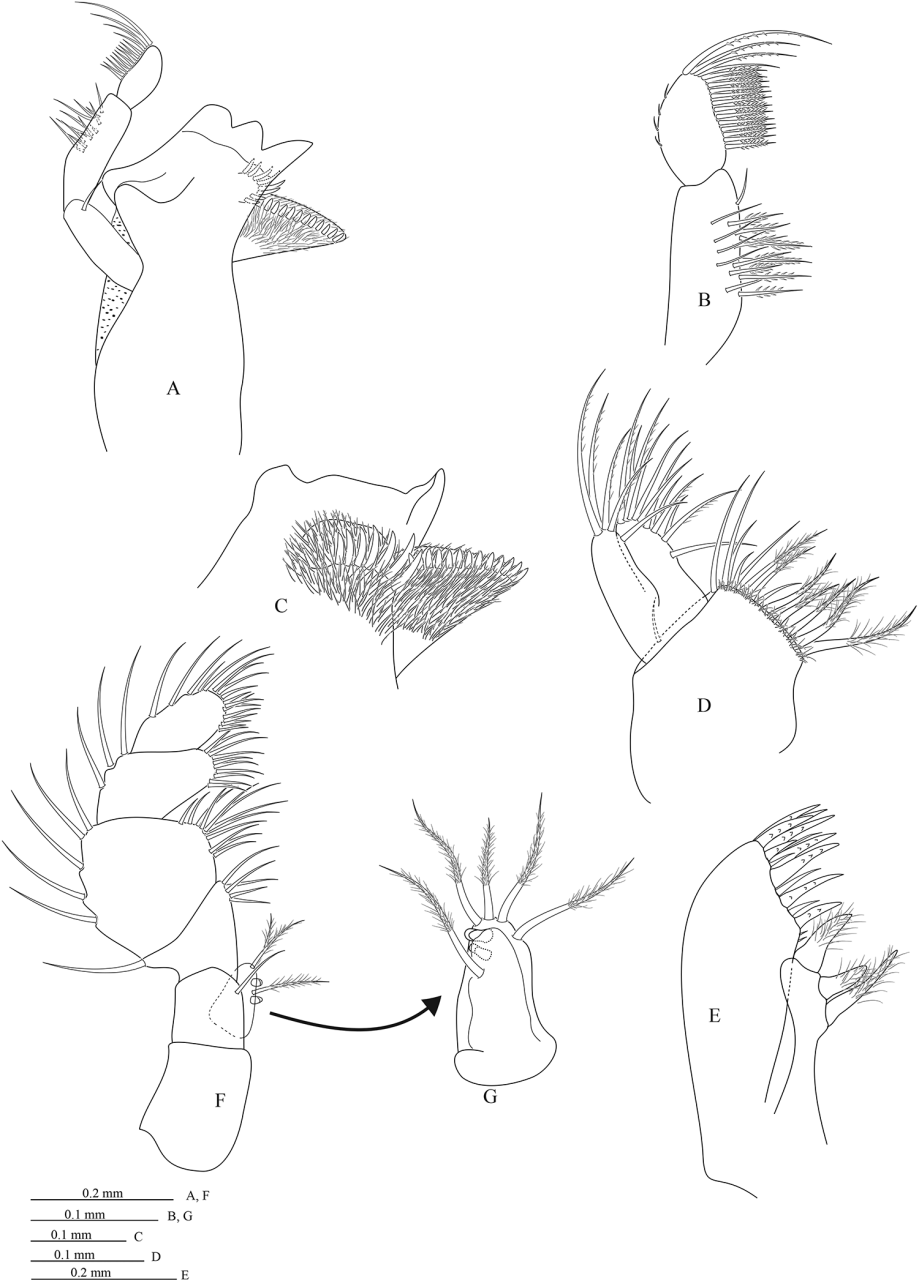
*Cirolana phuketensis* sp. n., male paratype (PSUZC–CR0286-02) (5.1 mm) **A** right mandible **B** dorsal view of article 3 of right mandible palp **C** distal part of left mandible **D** right maxilla **E** right maxillula **F** right maxilliped **G** endite.

*Mandible molar process* (Figure 3A, C) anterior margin with 12 flat teeth; without proximal cluster of long simple setae; right mandible spine row composed of 8 spines, left with 7 spines; palp articles 2 with 14 distolateral setae; palp article 3 with 17 robust biserrate setae (Figure 3B); *Maxillula* (Figure 3E) mesial lobe with 3 large and circumplumose RS; lateral lobe with 12 RS (plus 1 slender seta). *Maxilla* (Figure 3D) lateral lobe with 5 long simple setae, middle lobe with 12 long simple setae, maxilla mesial lobe with 1 distal simple seta and 12 proximal simple and plumose setae. *Maxilliped palp* (Figure 3F) article 2 mesial margin with 5 slender setae, lateral margin distally with 1 slender setae; article 3 mesial margin with 12 slender setae, lateral margin with 5 slender setae; article 4 mesial margin with 15 slender setae, lateral margin with 3 slender setae; article 5 distal margin 16 setae, lateral margin with 4 setae; maxilliped endite with 5 long CPS and 2 coupling setae (both left and right).

**Figure 4.**
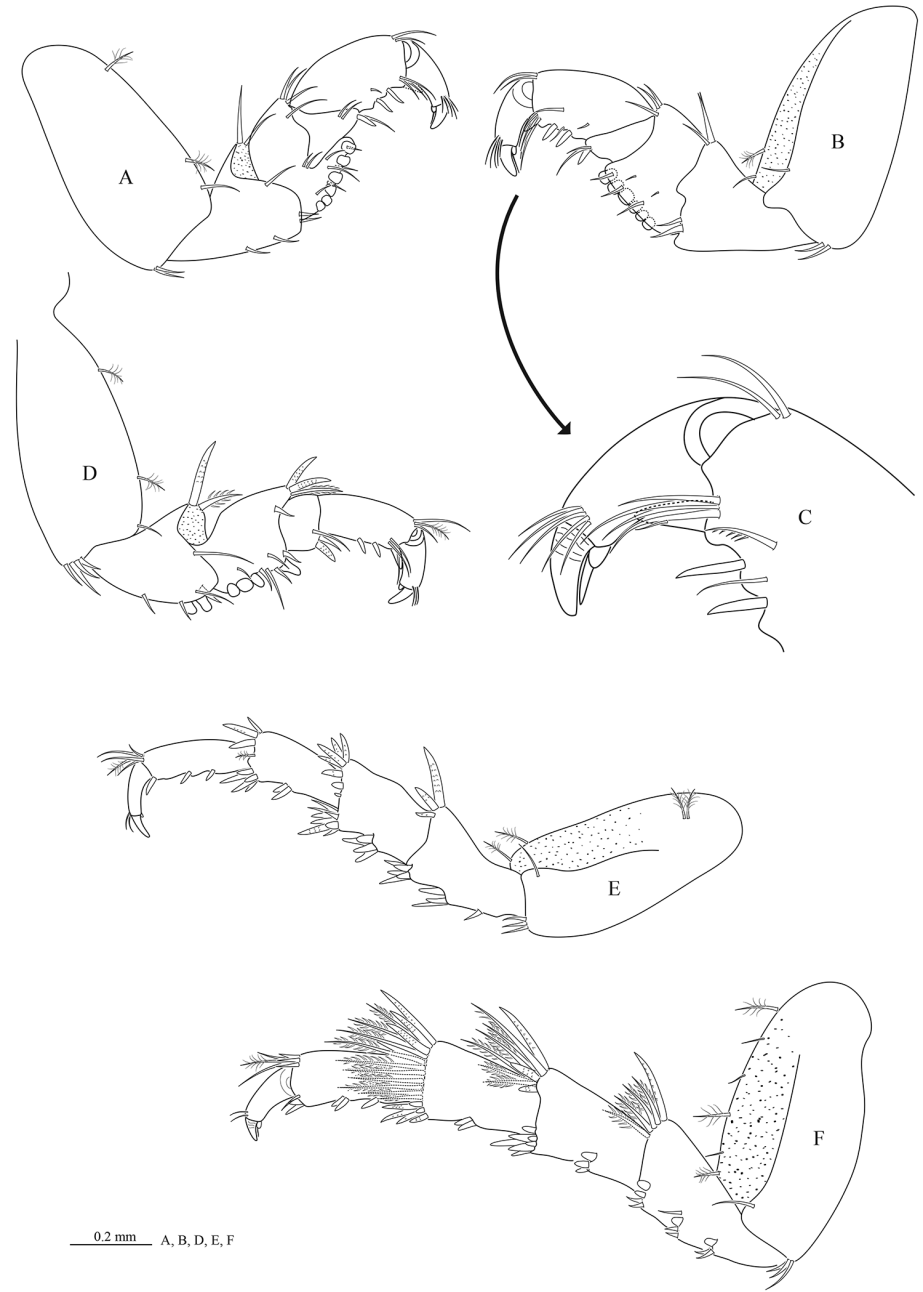
*Cirolana phuketensis* sp. n., male paratype (PSUZC–CR0286-02) (5.1 mm) **A** pereopod 1 **B** mesial view of pereopod 1 **C** mesial view of dactylus of pereopod 1 **D** pereopod 2 **E** pereopod 4 **F** pereopod 7.

*Pereopod 1* (Figure 4A, B) basis 2.0 times as long as greatest width, inferodistal angle with cluster of 2 acute setae; ischium 0.6 times as long as basis, inferior margin with 2 setae, superior distal margin with 2 RS; *merus* inferior margin with 6 molariform RS (set in row of 5 and 2), superior distal angle with 3 setae; *carpus* inferior margin with 1 RS (plus 1 slender seta); *propodus* 1.8 times as long as wide, inferior margin with 2 RS; *dactylus* (Figure 4C) 0.7 times as long as propodus; inferior margin lacking setal fringe. *Pereopod 2* (Figure 4D) *ischium* inferior margin with 2 stout, bluntly rounded RS, superior distal margin with 2 RS; *merus* inferior margin with 4 stout RS (set in row 3 and 1), superior distal margin with 3 acute RS; *carpus* inferodistal angle with 2 RS (plus 1 slender seta); *propodus* 2.3 times as long as wide, with 3 cluster of acute RS; *dactylus* 1.3 times as long as propodus. *Pereopod 3* similar to pereopod 2. *Pereopod 4* (Figure 4E) intermediate in form between pereopod 3 and pereopod 5. *Pereopod 6* similar to pereopod 7. Pereopod 7 (Figure 4F) *basis* 2.0 times as long as greatest width, superior margin convex, inferior margin with 3 palmate setae; *ischium* 0.6 times as long as basis, inferior margin with 7 RS (set in group 3 and 4), superior distal angle with 5 RS, inferior distal angle with 4 RS; *merus* 0.8 time as long as ischium, 1.5 times as long as wide, inferior margin with 3 RS, superior distal angle with 9 RS, inferior distal angle with 7 RS; *carpus* 0.8 time as long as ischium, 1.5 times as long as wide, inferior margin with 2 RS, superior distal angle with 17 RS, inferior distal angle with 10 RS; *propodus* 0.8 times as long as ischium, 2.3 times as long as wide, inferior margin with 3 clusters of RS (set in group 1 and 2), superior distal angle with 2 slender setae (plus 1 plumose seta and 3RS), inferior distal angle with 2 robust setae; *dactylus* 0.6 times as long as propodus.

*Penes* (Figure 2H) two low tubercles separated by 3% of sternal width.

**Figure 5.**
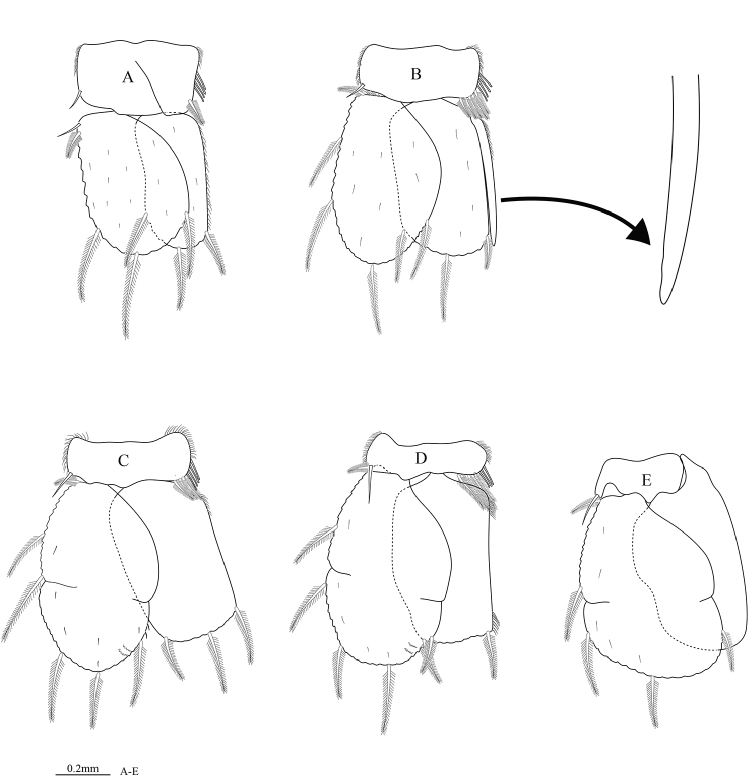
*Cirolana phuketensis* sp. n., male paratype (PSUZC–CR0286-02) (5.1 mm) (**A–E**) pleopods 1–5 respectively.

*Pleopod 1* (Figure 5A) exopod 1.4 times as long as wide, lateral margin straight, distally broadly rounded, mesial margin strongly convex, with 28 PMS from distal one-third; endopod 2.1 times as long as wide, distally broadly rounded, lateral margin strongly concave, with 15 PMS on distal margin only; peduncle 1.6 times as wide as long, mesial margin with 4 coupling hook. *Pleopod 2* (Figure 5B) exopod with 38 PMS, endopod with 14 PMS; appendix masculina with parallel margins, 1.0 times as long as endopod, distally narrowly rounded. *Pleopod 3* (Figure 5C) exopod with 39 PMS, endopod with 11 PMS. *Pleopod 4* (Figure 5D) exopod with 40 PMS, endopod with 10 PMS. *Pleopod 5* (Figure 5E) exopod with 38 PMS. *Pleopods* 2–5 peduncle distolateral margin with prominent acute RS.

**Figure 6.**
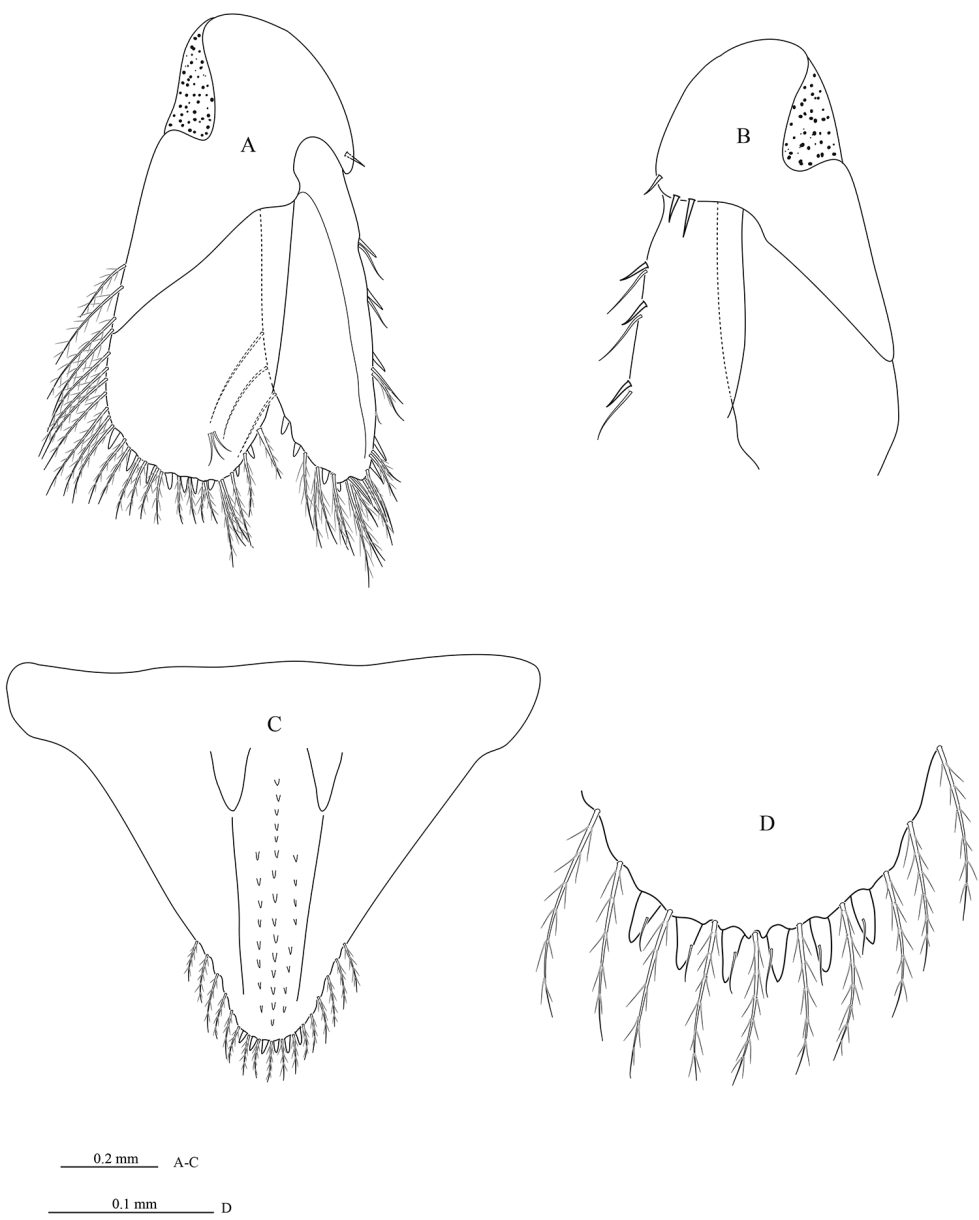
*Cirolana phuketensis* sp. n., male paratype (PSUZC–CR0286-02) (5.1 mm) **A** uropod **B** ventral view of uropod peduncle and exopod **C** pleotelson **D** pleotelson apex.

*Uropod* peduncle (Figure 6A) ventrolateral margin with 2 RS (Figure 6B), lateral margin with medial short acute RS, posterior lobe about one-half as long as endopod; rami extending beyond pleotelson, marginal setae in single tier. *Endopod* apically not bifid, broadly round, lateral margin straight, without prominent excision, with 2 RS, mesial margin strongly convex, with 7 RS. *Exopod* extending beyond end of endopod, 2.4 times as long as greatest width, apically not bifid, notched, lateral margin straight, with 5 RS, mesial margin weakly convex, with 4 RS.

##### Female (non-ovigerous).

Pereonite 7 without transverse row of tubercles. Pleonites 4–5 and pleotelson with low tubercles.

##### Size.

Adult males (n = 13) 4.6–5.8 mm (mean 5.1 mm); females (n = 16) 4.6–5.7 mm (mean 5.1 mm).

##### Variation.

Pleotelson (n = 28 [12 ♂ and 16♀]) with 5–6 RS, with 6 RS (3+3) most frequent (92%). Uropod endopod mesial margin with 6–7 RS, with 6 (82%) and 8 (4%) occurring only once, lateral margin with 1 RS (96%) and 2 (4%) occurring only once; exopod mesial margin with 2–4 RS, with 4 most frequent (92%), 2 and 3 occurring only once (3%), lateral margin with 5– 6 RS, with 5 most frequent (92%).

##### Remarks.

*Cirolana phuketensis* sp. n. is characterized by pereonites 2–4 each with a single transverse suture; pereonites 5–6 each with 2 transverse sutures; pereonites 7 with 3 transverse sutures that also form a nodulose ridge; antennula peduncular articles 1 and 2 fused; pleotelson dorsal surface with 2 sub-median longitudinal carinae, each of which has one prominent anterior tubercle, lateral margin weakly convex and posterior margin narrow rounded; pereopod 1 merus inferior margin with 6 molariform RS; penes in the form of two low tubercles.

*Cirolana phuketensis* sp. n. belongs to a group of species within *Cirolana* that is characterised by dorsal is characterised by dorsal nodular ornamentation on the pereon, pleon and pleotelson (Bruce 1986). This group of species has few widely separated robust setae on the uropodal exopod lateral margin, and the pleotelson posterior margin is truncate to narrowly rounded and has 6 or 8 robust seta; there is often clear sexual dimorphism in this group of species, with females more weakly ornamented that males, and dimorphic uropod shape.

In the South-East Asian region there are few similar species, although undescribed species are known. *Cirolana phuketensis* sp. n. differs from *C. tuberculata* from southern Philippines (see Delaney 1986), the only similar species in the region, by the pereon surface having tubercles on pereonite 7 (vs all pereonites lacking tubercles); coxae 4–7 with two oblique carinae (vs coxae 2–7 with single oblique carina); merus of pereopod 1 has 6 molariform robust setae (vs 4–5 molariform robust setae); pleonites 3–5 has tubercles (vs pleon smooth): uropodal exopod apex is notched (vs acute), lateral margin of exopod is straight (vs convex); lateral margin of endopod has two robust setae (vs four robust setae); dorsal surface of pleotelson with two tubercles and paired submedian longitudinal carina (vs parallel rows of four tubercles).

This species is also similar to *Cirolana grumula* Bruce, 1994 (Papua New Guinea) and the Australian species *Cirolana oreonota* Bruce, 1986. However, there are many characters that differentiate *Cirolana phuketensis* sp. n. from these species. *Cirolana phuketensis* sp. n. can be separated from *C. grumula* by having antennula articles 1 and 2 fused (vs unfused in *C. grumula*); pleotelson dorsal surface with 2 sub-median longitudinal carinae, each of which has one prominent tubercle (vs each of which has two prominent tubercles), pleotelson lateral margin weakly convex (vs straight) and posterior margin narrow rounded (vs subtruncate); pereopod 1 merus inferior margin has 6 molariform RS (vs 5 molariform RS); penes has 2 low tubercles (vs opening flush with surface of sternite 7. *Cirolana phuketensis* sp. n. differs from *C. oreonota* by pereonites 2–4 with transverse sutures (vs without transverse sutures on pereonites 2–4); pleotelson dorsal surface with two prominent ridges each with one anterior submedian tubercle (vs ridges not prominent each with 3 of submedian tubercles), pleotelson lateral margin weakly convex (vs straight), posterior margin narrow rounded (vs sub truncate) with 6 RS (vs 8 RS); antennal flagellum extending to middle of pereonite 4 (vs extending to anterior of pereonite 3); pereopod 1 merus inferior margin having 6 molariform RS (vs 5 molariform RS); penes 2 low tubercles (vs opening flush with surface of sternite 7).

##### Etymology.

The epithet is taken from the type locality.

**Figure 7.**
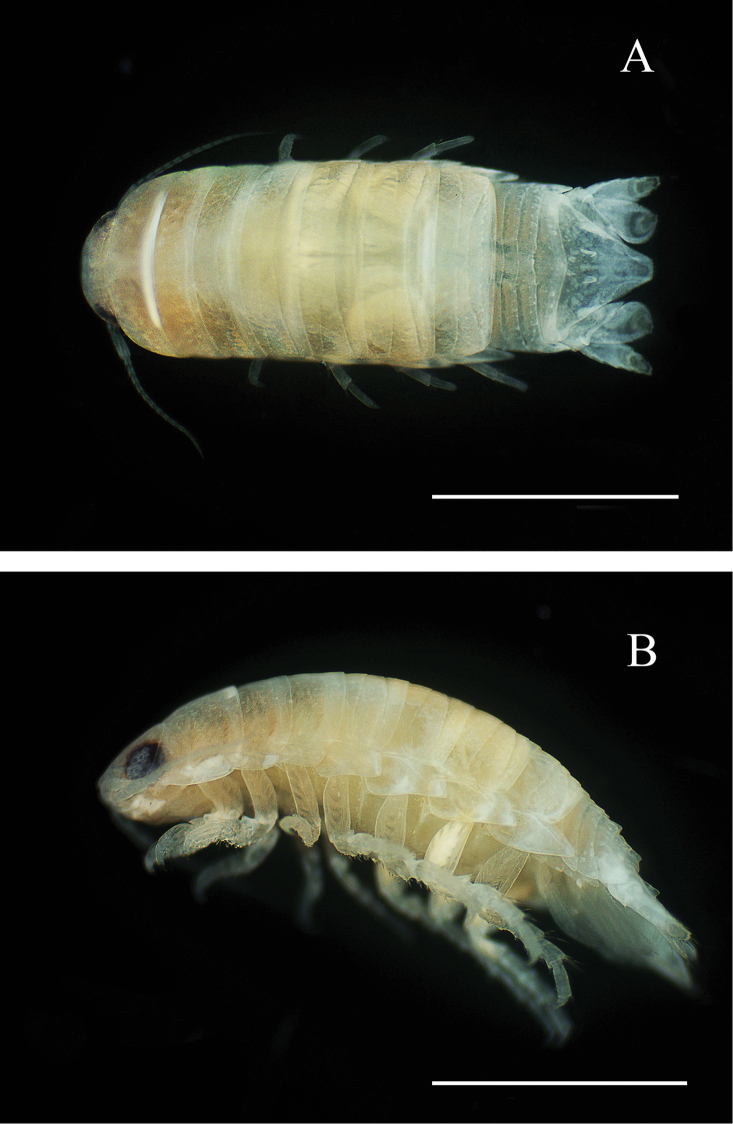
*Cirolana phuketensis* sp. n. male holotype, (PSUZC–CR0286-01) (5.0mm) **A** dorsal view **B** lateral view. Scale bar: 2 mm.

### Key to the species of *Cirolana* in Thailand

**Table:** 

1	Epimera of pleonites 3 and 4 not produced and medially indented	***C. rachanoi***
–	Epimera of pleonites 3 and 4 produced and medially not indented	**2**
2	Body dorsal surfaces without tubercles; rostral point present, folded ventrally and posteriorly, in contact with frontal lamina; uropodal rami apically bifid	**3**
–	Body dorsal surfaces with tubercles; anterior margin of head with or without rostral point; uropodal rami apically not bifid	**7**
3	Posterior margin of pleotelson with 12–14 RS; endopod of pleopods 3–4 distinctly smaller than exopod, without marginal plumose setae	***C. songkhla***
–	Posterior margin of pleotelson with 6–10 RS; endopod of pleopods 3–4 about equal to or slightly smaller than exopod, with marginal plumose setae	**4**
4	Antennula peduncle with articles 1 and 2 fused; male pereopod 1 without setal fringe; penial opening separated by 3% of sternal width; uropod peduncle ventrolateral margin with 1 sensory seta	***C. andamanensis***
–	Antennula peduncle with 4 unfused articles; male pereopod 1 with setal fringe; penial opening separated by 12–13% of sternal width; uropod peduncle ventrolateral margin with 3 sensory setae	**5**
5	Pleonite 3 with posterolateral margins extending to but not beyond posterior margin of pleonite 5; pleopod 1 endopod with lateral margin concave, appendix masculina 1.4 times as long as pleopod endopod	***C. phangnga***
–	Pleonite 3 with posterolateral margins extending clearly beyond posterior margins of pleonites 4 and 5; pleopod 1 endopod with lateral margin straight, appendix masculina ≤ 1.6 times as long as pleopod endopod	**6**
6	Uropodal rami apices equally bifid; appendix masculina lateral curved , 1.6 times as long as pleopod endopod	***C. siamensis***
–	Uropodal rami apices with lateral process prominent; appendix masculina straight, 1.9 times as long as pleopod endopod	***C. thailandica***
7	Endopod of pleopods 3–4 distinctly smaller than exopod, without marginal plumose setae; found in brackish water habitats	**8**
–	Endopod of pleopods 3–4 with marginal plumose setae; about equal to or slightly smaller than exopod; found in marine habitats	**9**
8	Anterior margin of head without rostral point; frontal lamina anterior margin rounded	***C. fluviatilis***
–	Anterior margin of head with rostral point, folded ventrally and posteriorly, in contact with frontal lamina; frontal lamina pentagonal	***C. willeyi***
9	Head weakly produced and overriding the antennules/a bases; inferior margins of pereopods 6 and 7 serrate	***C. bruscai***
–	Head not produced and overriding the antennules/a bases; inferior margins of pereopods 6 and 7 not serrated	***C. phuketensis* sp. n.**

## Supplementary Material

XML Treatment for Cirolana

XML Treatment for Cirolana phuketensis

## References

[B1] Ariyama H Angsupanich S Rodcharoen E (2010) Two New Species of the Genus Kamaka (Crustacea: Amphipoda: Kamakidae) from Songkhla Lagoon, Southern Thailand. Zootaxa 2404: 55–68.

[B2] Barnard KH (1936) Isopods collected by the R.I.MS. “Investigator”. Records of the Indian Museum 38(2): 147–191.

[B3] Brandt A Poore GCB (2003) Higher classification of the flabelliferan and related Isopoda based on a reappraisal of relationships. Invertebrate Systematics 17(6): 893–923. https://doi.org/10.1071/IS02032

[B4] Briggs JC (2000) Centrifugal speciation and centres of origin. Journal of Biogeography 27: 1183–1188. https://doi.org/10.1046/j.1365-2699.2000.00459.x

[B5] Briggs JC (2005) The marine East Indies: diversity and speciation. Journal of Biogeography 32: 1517–1522. https://doi.org/10.1111/j.1365-2699.2005.01266.x

[B6] Briggs JC Bowen BW (2013) Marine shelf habitat: biogeography and evolution. Journal of Biogeography 40: 1023–1035. https://doi.org/10.1111/jbi.12082

[B7] Bruce NL (1981) Cirolanidae (Crustacea: Isopoda) of Australia: Diagnoses of *Cirolana* Leach, *Metacirolana* Nierstrasz, *Neocirolana* Hale, *Anopsilana* Paulian & Debouteville, and three new genera—*Natatolana*, *Politolana* and *Cartetolana*. Australian Journal of Marine and Freshwater Research 32: 945–966. https://doi.org/10.1071/MF9810945

[B8] Bruce NL (1986) Cirolanidae (Crustacea: Isopoda) of Australia. Records of the Australian Museum, Supplement 6: 1–239. https://doi.org/10.3853/j.0812-7387.6.1986.98

[B9] Bruce NL (1994) *Cirolana* and related marine isopod crustacean genera (family Cirolanidae) from the coral reefs of Madang, Papua New Guinea. Cahiers de Biologie Marine 35: 375–413.

[B10] Bruce NL (2004a) New species of the *Cirolana* “*parva*-group” (Crustacea: Isopoda: Cirolanidae) from coastal habitats around New Zealand. Species Diversity 9: 47–66.

[B11] Bruce NL (2004b) *Cirolana mercuryi* sp. nov., a distinctive cirolanid isopod (Flabellifera) from the corals reefs of Zanzibar, East Africa. Crustaceana 76(9): 1071–1081. https://doi.org/10.1163/156854003322753420

[B12] Bruce NL Berggren M Bussawarit S (Eds) (2002) Proceedings of the International Workshop on the Crustacea in the Andaman Sea, Phuket Marine Biological Center, 29 November–20 December 1998. Phuket Marine Biology Center, Phuket, 532 pp.

[B13] Bruce NL Brandt A (2006) A new species of *Cirolana* Leach, 1818 (Crustacea, Isopoda, Cirolanidae) from the western Ross Sea, Antarctica, the first record of the genus from polar waters, Zoosystema 28(2): 315–324.

[B14] Bruce NL Olesen J (2002) Cirolanid Isopods from The Andaman Sea off Phuket, Thailand, with description of two new species. In: Bruce NL, Berggren M, Bussawarit S (Eds) Proceedings of the International Workshop on the Crustacea in the Andaman Sea, Phuket Marine Biological Center, 29 November–20 December 1998, Phuket Marine Biological Center Special Publication, 23, Phuket Marine Biological Center, Phuket, 109–131.

[B15] Bruce NL Schotte M (2015) Cirolanidae In: Schotte M, Boyko CB, Bruce NL, Poore GCB, Taiti S, Wilson GDF (Eds) World marine, freshwater and terrestrial isopod crustaceans database at http://www.marinespecies.org/aphia.php?p=taxdetails&id=118399

[B16] Bruce NL Wong HPS (2015) An overview of the marine Isopoda (Crustacea) of Singapore. Raffles Bulletin of Zoology, Supplement 31: 1–17.

[B17] Brusca RC Wetzer R France SC (1995) Cirolanidae (Crustacea: Isopoda: Flabellifera) of the Tropical Eastern Pacific. Proceedings of the San Diego Natural History Museum 30: 1–96.

[B18] Carpenter KE Barber PH Crandall ED Ablan-Lagma MA Ambariyanto G Ngurah Mahardika G Manjaji-Matsumoto BM Juinio-Menes MA Santos MD Starger CJ Toha AHA (2011) Comparative Phylogeography of the Coral Triangle and Implications for Marine Management, Journal of Marine Biology 2011: 1–14. https://doi.org/10.1155/2011/396982

[B19] Coleman CO Lowry JK Macfarlane T (2010) DELTA for beginners. An introduction into the taxonomy software package DELTA. Zookeys 45: 1–75. https://doi.org/10.3897/zookeys.45.263

[B20] Dallwitz MJ (1980) A general system for coding taxonomic descriptions. Taxon, 20: 41–46. https://doi.org/10.2307/1219595

[B21] Dallwitz MJ Paine TA Zurcher EJ (2006) User’s guide to the DELTA system: a general system for processing taxonomic descriptions. Available from: http://delta-intkey.com/ [accessed 15 October 2015]

[B22] Dallwitz MJ Paine TA Zurcher EJ (1997) User’s guide to the DELTA system. A general system for processing taxonomic descriptions. CSIRO Division of Entomology, Canberra.

[B23] Delaney PM (1986) The Synonymy of *Cirolana tuberculata* (Richardson, 1910) (Isopoda, Flabellifera, Cirolanidae). Proceedings of the Biological Society of Washington 99(4): 731–734.

[B24] Keable SJ (1995) Structure of the marine invertebrate scavenging guild of a tropical reef ecosystem: field studies at Lizard Island, Queensland, Australia. Journal of Natural History 29: 27–45. https://doi.org/10.1080/00222939500770021

[B25] Kensley B Schotte M (1989) Guide to the marine isopod crustaceans of the Caribbean. Smithsonian Institution Press. Washington, DC and London.

[B26] Kensley B (2001) Biogeography of the marine Isopoda of the Indian Ocean, with a check-list of species and records. In: Kensley B Brusca RC (Eds) Isopod systematics and evolution. Crustacean Issues 13: 205–264.

[B27] Leach WE (1818) Cymothoadés. In: Cuvier F (Ed.) Dictionnaire des sciences naturelles. Paris and Strasbourg 12: 338–354.

[B28] Richardson H (1910) Marine isopods collected in the Philippines by the U.S. Fisheries steamer *Albatross* in 1907–8. Washington D.C. Department of Commerce Bureau of Fisheries. Document No. 736.

[B29] Rodcharoen E Bruce NL Pholpunthin P (2014) *Cirolana songkhla*, a new species of brackish–water cirolanid isopod (Crustacea, Isopoda, Cirolanidae) from the lower Gulf of Thailand. ZooKeys 375: 1–14. https://doi.org/10.3897/zookeys.375.6573 10.3897/zookeys.375.6573 PMC3921561 24526843

[B30] Rodcharoen E Bruce NL Pholpunthin P (2016) Description of four new species of the *Cirolana* ‘*parva* group’ (Crustacea: Isopoda: Cirolanidae) from Thailand, with supporting molecular (COI) data. Journal of Natural History 50 (NOS31-32): 1935–1981 https://doi.org/10.1080/00222933.2016.1180718

[B31] Sidabalok CM (2013) List of marine isopods recorded from Indonesian waters. Marine Research in Indonesia 38(1): 49–66. https://doi.org/10.14203/mri.v38i1.56

[B32] Sidabalok C Bruce NL (2015) Revision of the cirolanid isopod genus *Odysseylana* Malyutina, 1995 (Crustacea) with description of two new species from Singapore. Zootaxa 4021(2): 351–367. https://doi.org/10.11646/zootaxa.4021.2.6 26624133 10.11646/zootaxa.4021.2.6

[B33] Sidabalok CM Bruce NL (2016) Redescription of three cirolanid isopods (Crustacea: Peracarida) from Indonesia. Zootaxa 414(3): 277–290. https://doi.org/10.11646/zootaxa.4114.3.4 10.11646/zootaxa.4114.3.4 27395130

[B34] Sidabalok C Bruce NL (2017a) Review of the species of the *Cirolana* ‘*parva*-group’ (Cirolanidae: Isopoda: Crustacea) in Indonesian and Singaporean waters Zootaxa [in press].

[B35] Sidabalok C Bruce NL (2017b) Review of the *Cirolana* ‘*pleonastica*-group’ (Crustacea: Isopoda: Cirolanidae) with description of four new species from the Indo-Malaysian region. Raffles Bulletin of Zoology [submitted].

[B36] Sidabalok C Bruce NL (2017c) *Cirolana bambang*, a distinctive new species of *Cirolana* Leach, 1818 (Cirolanidae: Isopoda: Crustacea) from Bitung, Indonesia. Zootaxa xxx(submitted). 10.11646/zootaxa.4375.3.10 29690082

[B37] Suvatti C (1967) Fauna of Thailand (2^nd^ed.). Applied Scientific Research Corporation of Thailand, Bangkok, 143–17.

[B38] Svavarsson J (2002) Gnathiidae (Crustacea, Isopoda) from the Andaman Sea, Thailand: new records and a new species. Phuket Marine Biological Center Special Publication 23(1): 149–156.

[B39] Svavarsson J (2002) *Elaphognathia korachaensis* sp. nov., a new gnathiid species (Crustacea, Isopoda) from Thailand. Phuket Marine Biological Center Special Publication 23(1): 157–164.

[B40] Wongkamhaeng K Coleman CO Azman BAR (2013) *Maeropsis paphavasitae* and *Rotomelita longipropoda*, two new species (Crustacea, Amphipoda) from Lower Gulf of Thailand. ZooKeys 307: 15–33 (2013). https://doi.org/10.3897/zookeys.307.5273 10.3897/zookeys.307.5273 PMC3689061 23794921

[B41] Wongkamhaeng K Azman BAR Puttapreecha R (2012a) *Cheiriphotis trifurcata*, new species (Crustacea, Amphipoda, Corophiidae, Protomedeiinae) from the Seagrass Bed of the Lower Gulf of Thailand. ZooKeys 187: 71–89. https://doi.org/10.3897/zookeys.187.3219 10.3897/zookeys.187.3219 PMC3345904 22577331

[B42] Wongkamhaeng K Pattaratumrong MS Puttapreecha R (2014) Melitid amphipods from the Gulf of Thailand, with a description of *Dulichiella pattaniensis*, a new species. ZooKeys 408: 1–18. https://doi.org/10.3897/zookeys.408.7292 10.3897/zookeys.408.7292 PMC4042822 24899833

[B43] Wongkamhaeng K Pholpunthin P Azman BAR (2012b) *Grandidierella Halophilus* a new species of the family Aoridae (Crustacea: Amphipoda) from the saltpans of The Inner Gulf of Thailand. The Raffles Bulletin of Zoology 60(2): 433–447.

